# Incidence, risk factors, and clinical outcomes of HBV reactivation in non-liver solid organ transplant recipients with resolved HBV infection: A systematic review and meta-analysis

**DOI:** 10.1371/journal.pmed.1004196

**Published:** 2023-03-15

**Authors:** Saifu Yin, Fan Zhang, Jiapei Wu, Tao Lin, Xianding Wang

**Affiliations:** 1 Department of Urology/Institute of Urology, West China Hospital, Sichuan University, Chengdu City, Sichuan Province, China; 2 Organ Transplantation Center, West China Hospital, Sichuan University, Chengdu City, Sichuan Province, China; University of Texas Southwestern Medical Center, UNITED STATES

## Abstract

**Background:**

Current guidelines do not recommend routine antiviral prophylaxis to prevent hepatitis B virus (HBV) reactivation in non-liver solid organ transplant (SOT) recipients with resolved HBV infection, even in anti-hepatitis B surface antigen (anti-HBs)-negative recipients and those receiving intense immunosuppression. This systematic review and meta-analysis aimed to determine the incidence, risk factors, and clinical outcomes of HBV reactivation in non-liver SOT recipients.

**Methods and findings:**

Three databases (PubMed, Embase, and Cochrane Library) were systematically searched up to December 31, 2022. Clinical studies reporting HBV reactivation in non-liver SOT recipients were included. Case reports, case series, and cohort studies with a sample size of less than 10 patients were excluded. Random-effects analysis was used for all meta-analyses. We included 2,913 non-liver SOT recipients with resolved HBV infection from 16 retrospective cohort studies in the analysis. The overall HBV reactivation rate was 2.5% (76/2,913; 95% confidence interval [95% CI 1.6%, 3.6%]; I^2^ = 55.0%). Higher rates of reactivation were observed in recipients with negative anti-HBs (34/421; 7.8%; 95% CI [5.2%, 10.9%]; I^2^ = 36.0%) by pooling 6 studies, experiencing acute rejection (13/266; 5.8%; 95% CI [2.3%, 14.5%]; I^2^ = 63.2%) by pooling 3 studies, receiving ABO blood type-incompatible transplantation (8/111; 7.0%; 95% CI [2.9%, 12.7%]; I^2^ = 0%) by pooling 3 studies, receiving rituximab (10/133; 7.3%; 95% CI [3.4%, 12.6%]; I^2^ = 0%) by pooling 3 studies, and receiving anti-thymocyte immunoglobulin (ATG, 25/504; 4.9%; 95% CI [2.5%, 8.1%]; I^2^ = 49.0%) by pooling 4 studies. Among recipients with post-transplant HBV reactivation, 11.0% (7/52; 95% CI [4.0%, 20.8%]; I^2^ = 0.3%) developed HBV-related hepatic failure, and 11.0% (7/52; 95% CI [4.0%, 20.8%]; I^2^ = 0.3%) had HBV-related death. Negative anti-HBs (crude odds ratio [OR] 5.05; 95% CI [2.83, 9.00]; *p* < 0.001; I^2^ = 0%), ABO blood type-incompatible transplantation (crude OR 2.62; 95% CI [1.05, 6.04]; *p* = 0.040; I^2^ = 0%), history of acute rejection (crude OR 2.37; 95% CI [1.13, 4.97]; *p* = 0.022; I^2^ = 0%), ATG use (crude OR 3.19; 95% CI [1.48, 6.87]; *p* = 0.003; I^2^ = 0%), and rituximab use (crude OR 3.16; 95% CI [1.24, 8.06]; *p* = 0.016; I^2^ = 0%) increased the risk of reactivation. Adjusted analyses reported similar results. Limitations include moderate heterogeneity in the meta-analyses and that most studies were conducted in kidney transplant recipients.

**Conclusions:**

Non-liver SOT recipients with resolved HBV infection have a high risk of HBV-related hepatic failure and HBV-related death if HBV reactivation occurs. Potential risk factors for HBV reactivation include rituximab use, anti-thymocyte immunoglobulin use, anti-HBs negative status, acute rejection history, and ABO blood type-incompatible transplantation. Further research on monitoring and routine antiviral prophylaxis of non-liver SOT recipients at higher risk of HBV reactivation is required.

## Abstract 2

In a systematic review and meta-analysis, Tao Lin and colleagues investigate the incidence, risk factors and clinical outcomes of HBV reactivation in non-liver solid organ transplant recipients with resolved HBV infection.

## Author summary

**Why was this study done?:**

- Approximately 2 billion people globally have serologic evidence of resolved hepatitis B virus (HBV) infection, characterized by positive antibody against hepatitis B core antigen. In the context of solid organ transplantation, immunosuppression can result in HBV reactivation in patients with resolved HBV infection.
- Current guidelines consider that it is unnecessary to take antiviral prophylaxes to prevent HBV reactivation in non-liver solid organ transplant (SOT) recipients with resolved HBV infection, even in anti-hepatitis B surface antigen (anti-HBs)-negative recipients and those receiving intense immunosuppression.
- To our knowledge, the incidence, risk factors, and clinical outcomes after HBV reactivation are unknown for non-liver SOT recipients because no reviews of previous studies have been published.

**What did the researchers do and find?:**

- We conducted a systematic review and meta-analysis. We screened 2,329 publications and included 16 articles in the final meta-analysis. The incidence of HBV reactivation was 2.5%, but differed according to patient characteristics.
- Individuals with HBV reactivation may have a high incidence of impaired liver function, hepatic cirrhosis, HBV-related hepatic failure, and HBV-related death.
- Anti-thymocyte immunoglobulin use, rituximab use, anti-HBs negative status, acute rejection history, and ABO blood type-incompatible transplantation were potential risk factors of HBV reactivation.

**What do these findings mean?:**

- Our study reported the incidence and potential risk factors for HBV reactivation, and its complications, after transplantation.
- Our results suggest that monitoring and routine antiviral prophylaxis in non-liver SOT recipients at high risk of HBV reactivation could be beneficial. Further research is required to determine the impact on outcomes after transplantation.

## Introduction

Hepatitis B virus (HBV) infection is a major public health concern and the main cause of infection-related deaths worldwide [1,2]. Globally, approximately 2 billion people have serologic evidence of resolved HBV infection, defined as undetectable serum HBV DNA and negative HB surface antigen (HBsAg) but positive antibody against HB core antigen (anti-HBc). In such population, HBV virions are cleared, while the HBV nucleocapsid is transported into the nucleus and generates closed circular (ccc) DNA. In solid organ transplant (SOT) recipients, immunosuppression can result in HBV reactivation with ccc DNA serving as the replication template, which may further cause progressive hepatitis, hepatic failure, and even death [3].

For liver transplant (LT) recipients receiving grafts from donors with resolved HBV infection, reported HBV reactivation rates vary from 4% to 58%, depending on recipients’ HBV serological status and antiviral prophylaxis regimens [4,5]. Based on European Association for the Study of the Liver (EASL) 2017 Clinical Practice Guidelines on the Management of Hepatitis B Virus Infection, these LT recipients are recommended to be given lifelong HBV prophylaxis and routine HBV serological monitoring (strong recommendation) [6]. In non-liver SOT recipients with resolved HBV infection, the native liver is not surgically removed, potentially resulting in HBV reactivation in the setting of immunosuppression. Different from LT recipients, published studies have reported a low incidence of HBV reactivation in non-liver SOT recipients, estimated at 1% to 3% [7,8]. Considering the relatively low risk, 2020 Kidney Diseases Improving Global Outcomes (KDIGO) Clinical Practice Guideline on the Evaluation and Management of Candidates for Kidney Transplantation recommends no routine monitoring and no antiviral prophylaxis in patients with resolved HBV infection after transplantation [9].

However, the level of evidence to support this recommendation was very low (1D, meaning that the estimate of effect is very uncertain) [9]. Furthermore, with the development of highly sensitized transplantation (e.g., human leukocyte antigen [HLA]-incompatible, ABO blood type-incompatible transplantation), more intensive immunosuppression is required, including B- and T-lymphocyte depleting agents (e.g., rituximab, bortezomib, anti-thymocyte immunoglobulin [ATG]) [10,11]. In this setting, the immune system is further weakened, placing these recipients at a higher risk of HBV reactivation. According to the American Gastroenterological Association (AGA) Institute and Asian-Pacific Association for the Study of the Liver (APASL) guidelines on hepatitis B reactivation related to the use of immunosuppressive therapy, patients receiving rituximab are at high risk of HBV reactivation (>10%) [12,13]. In these patients, both routine antiviral prophylaxis and HBV serology monitoring are recommended (strong recommendation). In contrast, for patients at low risk of HBV reactivation, guidelines have suggested against antiviral prophylaxis but recommended serological monitoring (weak recommendation) [12,13]. Additionally, although uncommon, severe hepatitis, HBV-related hepatic failure, and HBV-related death have been reported following HBV reactivation under immunosuppression [14,15].

In this study, we conducted a systematic review and meta-analysis to summarize the incidence, risk factors, and clinical outcomes of HBV reactivation in non-liver SOT recipients with resolved HBV infection. These results could help identify patients at high risk of HBV reactivation and provide guidance for HBV serological monitoring and posttransplant antiviral prophylaxis in clinical practice.

## Methods

This systematic review and meta-analysis was registered in PROSPERO (CRD42021278024) [16] and was conducted based on the Preferred Reporting Items for Systematic Reviews and Meta-Analyses (PRISMA) guidelines [17].

### Search strategy

We conducted systematic searches in PubMed (June 1997 to August 15, 2021), Embase (1974 to August 15, 2021), and Cochrane Library (database inception to August 15, 2021), by combining MeSH terms and keywords of the following terms: “hepatitis b virus,” “HBsAg,” “antibody against hepatitis B surface antigen (anti-HBs),” “hepatitis B e antigen,” “hepatitis B virus e antibody,” “anti-HBc,” “solid organ transplantation,” “kidney transplantation,” “lung transplantation,” “heart transplantation,” and “pancreas transplantation.” An updated search was conducted up to December 31, 2022. The search strategy is listed in S1 Table. There were no language limitations to this study. References from review articles and eligible studies were also reviewed for additional eligible studies.

### Inclusion and exclusion criteria

The inclusion criteria were as follows: (i) retrospective or prospective studies; (ii) resolved HBV infection, defined as positive anti-HBc but undetectable serum HBV DNA and negative HBsAg; and (iii) kidney, lung, heart, pancreas, or multi-non-liver organ transplantation. Case reports, case series, and cohort studies with less than 10 patients were excluded. Studies that only included LT recipients were excluded. Two authors (SFY and FZ) independently reviewed the titles and abstracts. Potential eligible studies were selected for full-text screening. Disagreements were resolved by consensus and agreement on study selection was evaluated using Cohen’s kappa.

### Data extraction and quality assessment

We extracted relevant information from eligible publications, including first author, location, study design, sample size, baseline characteristics (age, sex, type of transplantation, use of rituximab, use of ATG, proportion of anti-HBs-positive recipients, proportion of anti-HBc-positive donors, and proportion of combined hepatitis virus C [HCV] infection), incidence of HBV reactivation, time for HBV reactivation, incidence of hepatic cirrhosis, incidence of impaired liver function, incidence of HBV-related hepatic failure, incidence of HBV-related death, definition of resolved HBV infection, definition of HBV reactivation, follow-up period, and potential risk factors of HBV reactivation.

Two researchers (SFY and FZ) independently assessed the quality of observational studies using the Newcastle–Ottawa scale (NOS) [18]. The NOS evaluates the following criteria: selection, comparability, and outcome with a minimum score of 0 and a maximum score of 9. A score of 7 or more was reflective of high methodological quality, a score of 5 or 6 indicated moderate quality, and a score of 4 or less indicated low quality.

### Outcome measurements

The primary outcome was the incidence of HBV reactivation, defined as the reemergence of HBsAg (or HBsAg seroconversion) or HBV DNA, as specified by the authors. Considering the inconsistent demographic information, HBV serological status, and immunosuppression type, we predefined a subgroup analyses to identify those at a higher risk of HBV reactivation. Furthermore, we evaluated the impact of HBV reactivation on the incidence of impaired liver function, hepatic cirrhosis, HBV-related hepatic failure, and HBV-related death. Secondary outcome measures were potential risk factors for HBV reactivation. We explored the role of sex, location, anti-HBs status, rituximab use, ATG use, ABO blood type-incompatible transplantation, history of acute rejection, and posttransplant antiviral prophylaxis.

### Statistical analysis

Meta-analysis was performed using R statistical software version 4.0.0 (R Foundation for Statistical Computing, Vienna, Austria) with the package “meta” [19]. Random-effects analysis was used for all meta-analyses owing to the clinical heterogeneity inherent in the data and different sample sizes of the included studies.

We estimated the incidence of HBV reactivation using the inverse-variance method (DerSimonian and Laird) [20]. Because of the low number of events, arcsine transformation was applied to stabilize the variance of the risk estimates [21]. A 95% prediction interval was generated to predict where the next study might lie. Summary-level meta-regression was conducted when at least 3 data points were collected to explore potential sources of heterogeneity or prognostically relevant prespecified study-level covariates. Sensitivity analyses were performed by removing 1 study at a time and repeating the meta-analysis to evaluate the stability of the results. Small study effects were evaluated using a funnel plot with Egger’s test. Heterogeneity between estimates was assessed using the *I*^2^ statistic: 0% to 30%, mild heterogeneity; 30% to 60%, moderate heterogeneity; and 60% to 100%, substantial heterogeneity. *P*-values less than 0.05 were considered statistically significant.

## Results

**Fig 1:**
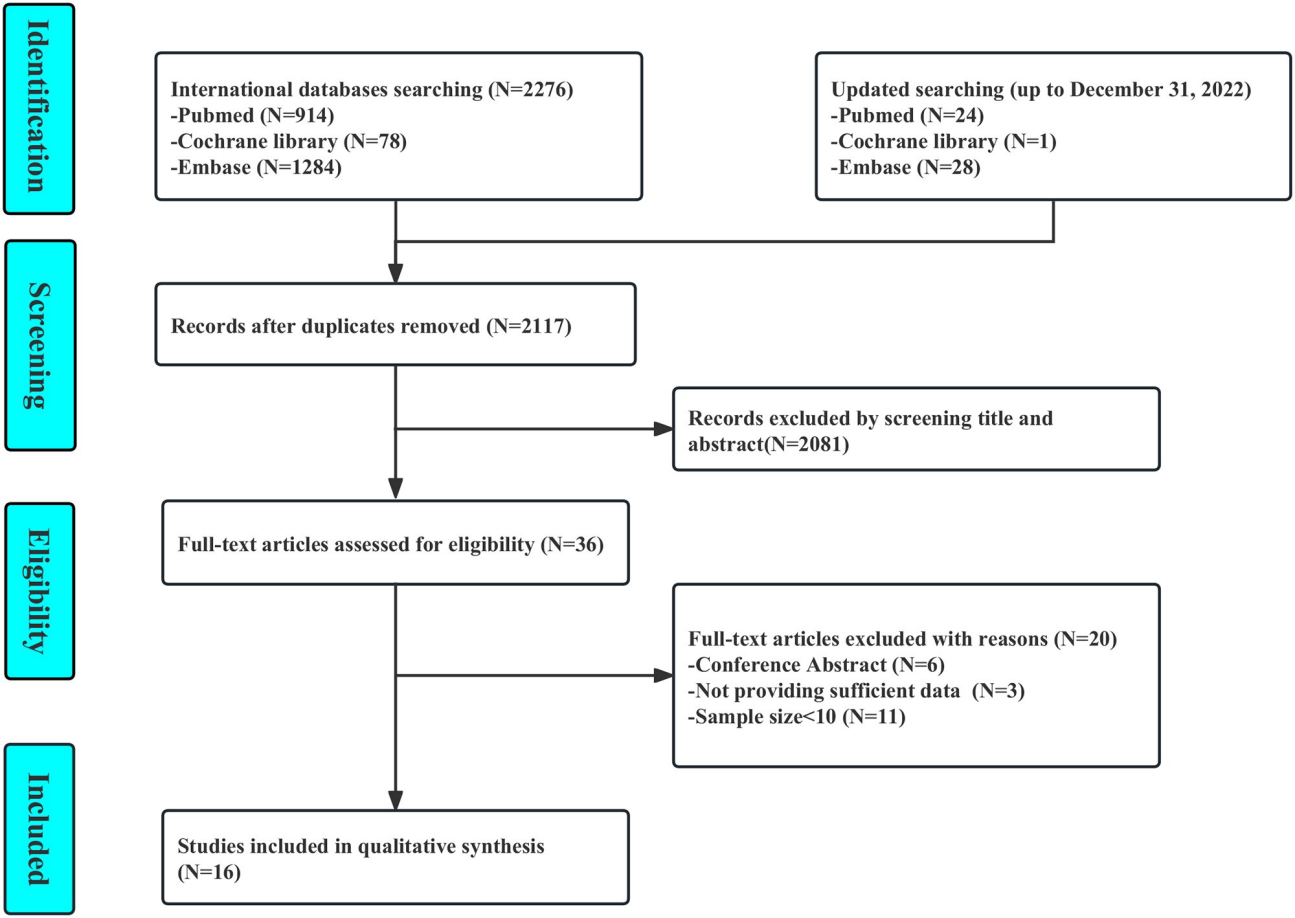
Selection process of included studies.

Our initial searches yielded 2,276 publications and updated searches yielded an additional 53 publications. After removing 212 duplicates, 2,117 were excluded based on title and abstract screening, and 20 were excluded after full-text screening (Fig 1). Finally, 16 studies were included in the final meta-analysis [7,8,14,15,22–33]. The inter-reviewer agreement was excellent, with a kappa statistic of 0.94.

### Characteristics of the included studies

The characteristics of the included studies are summarized in Table 1. The median/mean follow-up duration varied from 31.5 to 151 months. Seven studies were performed in Asia (4 in South Korea, 2 in Japan, and 1 in China), 2 in North America (both in the United States of America), and 7 in Europe (2 in Belgium, 2 in Portugal, 1 in Germany, 1 in Italy, and 1 in Spain). All the studies were retrospective, with sample sizes ranging from 11 to 951. Thirteen studies reported the incidence of HBV reactivation in kidney transplant recipients, 2 in non-liver SOT recipients, and 1 in heart transplant recipients. The mean/median age of the included patients ranged from 42.1 to 57.2 years. The quality of the cohort studies was assessed according to the NOS tool, which showed that 6 studies were of high quality, 8 were of moderate quality, and 2 were of low quality (S2 Table). Eight studies clearly reported that no posttransplant prophylaxis against HBV reactivation was administered (S3 Table), 3 studies included patients with or without prophylaxis, and 5 studies did not report the prophylaxis status.

**Table 1:** Characteristics of 16 included studies.

Author	Country	Affiliation	Study type	Solid organ type	Sample size	Age (years)	Sex, (Male, %)	ABO blood type-incompatible transplantation (n/total)	Anti-thymocyte globulin (n/total)	Rituximab (n/total)	HBV vaccination (n/total)	Donor anti-HBc- positive (n/total)	Anti-HBs positive (n/total)	HBV prophylaxis (n/total)	Follow-up
Shaikh 2022 [33]	USA	University of Southern California	Retrospective cohort	Kidney	161	Mean: 59.5	NA	NA	122/161	NA	42/161	NA	128/161	14/161	Median: 1,136.0 days
Mei 2020 [31]	Japan	Kyushu University Hospital	Retrospective cohort	Kidney	52	Mean: 57.2	63.5%	14/52	NA	18/52	0/52	NA	NA	NA	Mean: 41.5 months
Kim 2020 [30]	South Korea	Samsung Medical Center, Seoul, South Korea	Retrospective cohort	Kidney	449	Median: 51	63.2%	92/449	207/449	66/449	NA	133/499	389/449	0/449	Median: 6.7 years
Alvarez-Lopez 2020 [29]	Spain	NA	Retrospective cohort	Solid (non-liver)	40	Mean: 54	63.0%	NA	NA	0	NA	NA	NA	0/40	NA
Querido 2019 [28]	Portugal	Hospital de Santa Cruz	Retrospective cohort	Kidney	70	Mean: 51.9	71.4%	NA	NA	11/70	NA	NA	64/70	0/70	Median: 151 months
Meng 2018 [27]	Portugal	University of Campania	Retrospective cohort	Kidney	95	Median: 50	34.6%	NA	21/95	0	NA	NA	86/95	0/95	Mean: 93 months
Lee 2018 [26]	South Korea	Yonsei University Health System	Retrospective cohort	Kidney	336	Mean: 50.6	65.2%	NA	41/336	91/336	NA	112/336	289/336	NA	Median: 74 months
Jeon 2018 [15]	South Korea	Asan Medical Center	Retrospective cohort	Kidney	951	Mean: 42.1	56.5%	55/951	NA	157/951	NA	374/951	809/951	NA	Median: 114 months
Vitrone 2017 [25]	Italy	Piazzale Ettore Ruggieri snc	Retrospective cohort	Heart	11	Median: 52	70.1%	NA	NA	NA	NA	NA	NA	0/11	Median: 35.5 months
Lee 2017 [24]	South Korea	Yonsei University Health System	Retrospective cohort	Kidney	172	Mean: 49.8	74.4%	31/172	18/172	49/172	NA	60/172	147/172	0/172	median: 58 months
Nishimura 2013 [23]	Japan	Hyogo Prefectural Nishinomiya Hospital	Retrospective cohort	Kidney	34	Mean: 48.4	55.9%	NA	NA	NA	NA	NA	NA	NA	Median: 57.1 months
Chen 2013 [14]	China	The First Affiliated Hospital of Sun Yat-sen University	Retrospective cohort	Kidney	322	36/322>60 years old	67.6%	NA	134/322	NA	NA	NA	180/322	110/322	Median: 8.6 years
Kanaan 2012 [22]	Belgium	Université Catholique de Louvain	Retrospective cohort	Kidney	93	Median: 56	61.0%	NA	NA	NA	NA	NA	74/93	0/93	Median: 73 years
Berger 2005 [32]	Germany	J.W. Goethe University Hospital	Retrospective cohort	Kidney	228	NA	NA	NA	NA	NA	NA	NA	NA	NA	NA
Duhart 2003 [8]	USA	university of Tennessee Health Science Center	Retrospective cohort	Solid (non-liver)	22	Mean: 46	59.1%	NA	11/22	NA	NA	4/22	18/22	1/22	3.1–71.4 years.
Blanpain 1998 [7]	Belgium	Hospital Erasme	Retrospective cohort	Kidney	49	NA	NA	NA	NA	NA	NA	NA	NA	0/49	NA

anti-HBc, antibody against hepatitis b core antigen; anti-HBs, antibody against hepatitis b surface antigen; HBV, hepatitis b virus; NA, not available.

### Incidence of HBV reactivation

**Fig 2:**
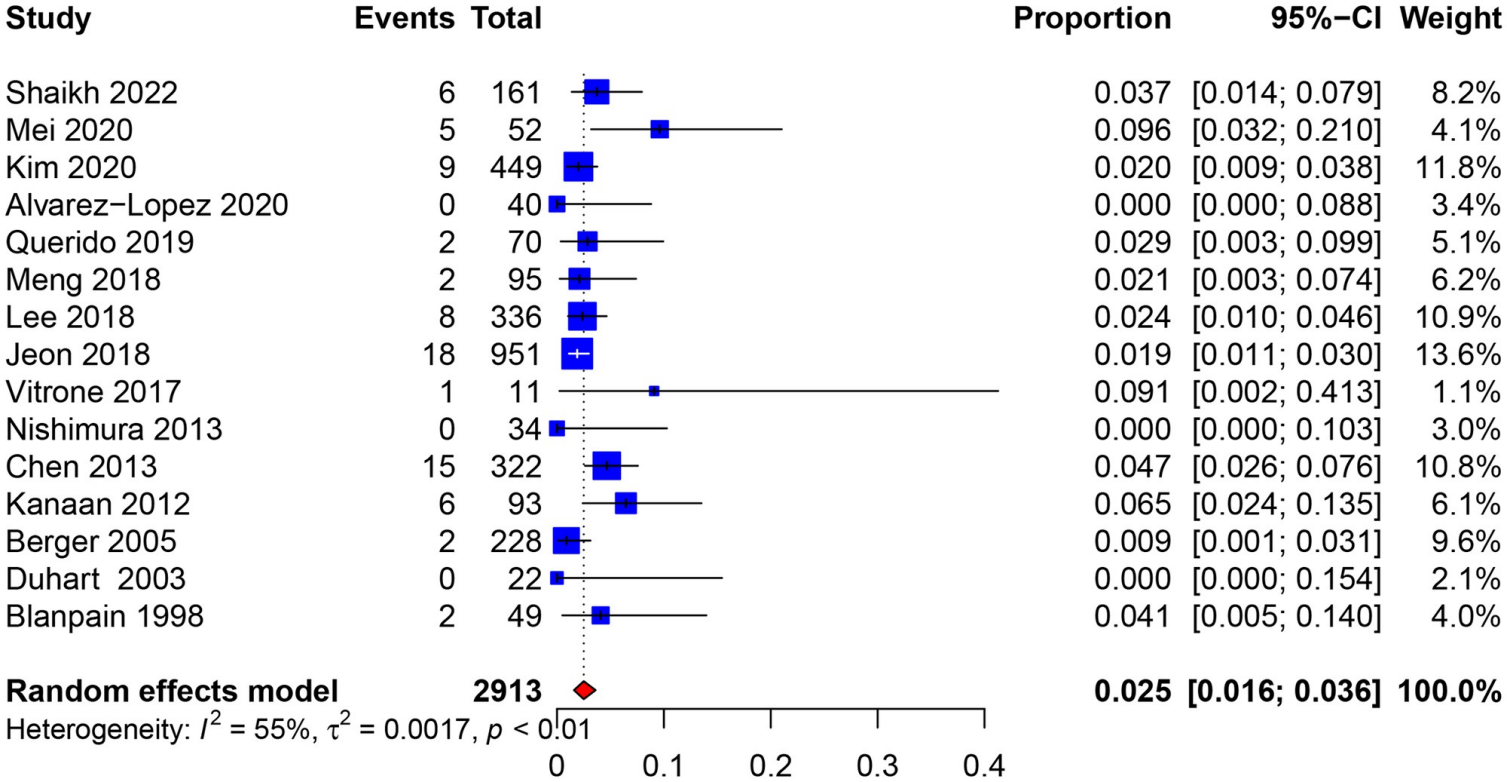
Pooled incidence of hepatitis b virus reactivation in non-liver solid organ transplant recipients with resolved infection. 95% CI, 95% confidence interval.

Among 2,913 patients with resolved HBV infection, 76 developed HBV reactivation (random-effects, 2.5%; 95% confidence interval [95% CI 1.6%, 3.6%]) (Fig 2). The time for HBV reactivation ranged from 5 months to 15 years posttransplant (Table 2). There was no evidence of small study effects (p _Egger_ = 0.780) (S1A Fig). Sensitivity analyses showed good stability of the pooled results (S1B Fig). Moderate heterogeneity indicated that baseline characteristics varied significantly among studies (I^2^ = 54.6%; p _heterogeneity_ = 0.006) (S4 Table). Meta-regression analyses indicated that age (*p* = 0.053), solid organ type (*p* = 0.077), rituximab use (*p* = 0.056), and the anti-HBs status of the recipient (*p* = 0.013) may be associated with the risk of HBV reactivation (S5 Table).

**Table 2:** Hepatitis b virus-related outcomes after reactivation among included studies.

Author	Sample size	HBV reactivation	HBV reactivation time	Hepatic cirrhosis	Liver function impairment	HBV-related hepatic failure	HBV-related death
Shaikh 2022 [33]	161	6	Median: 108.0 (IQR: 4.0–356.0) days for 5 recipients not receiving prophylaxis; 1,568 days for 1 recipient receiving prophylaxis	1	0	NA	NA
Mei 2020 [31]	52	5	NA	0	NA	0	0
Kim 2020 [30]	449	9	Median: 2.8 (IQR: 1.4–11.5) years	NA	1	NA	NA
Alvarez-Lopez 2020 [29]	40	0	NA	NA	NA	NA	NA
Querido 2019 [28]	70	2	41 months, 35 months	0	0	0	0
Meng 2018 [27]	95	2	NA	1	1	1	1
Lee 2018 [26]	336	8	Median: 11 months (range, 5–22 months) for 5 patients in standard-dose rituximab group; 48 months (range, 24–57 months) for 3 patients without rituximab	5	5	1	1
Jeon 2018 [15]	951	18	NA	NA	NA	2	2
Vitrone 2017 [25]	11	1	22 months	0	0	0	NA
Lee 2017 [24]	172	7	Median: 11 months (range: 5–22 months)	2	5	1	1
Nishimura 2013 [23]	34	0	NA	NA	NA	NA	NA
Chen 2013 [14]	322	15	Nine occurred within the first 6 months after kidney transplantation.	NA	12	2	2
Kanaan 2012 [22]	93	6	Three within the first posttransplant year.	NA	NA	NA	NA
Berger 2005 [32]	228	2	15 years, 5 years	NA	NA	NA	NA
Duhart 2003 [8]	22	0	NA	NA	NA	NA	NA
Blanpain 1998 [7]	49	2	NA	1	2	1	1

HBV, hepatitis b virus; IQR, interquartile range; NA, not available.

**Fig 3:**
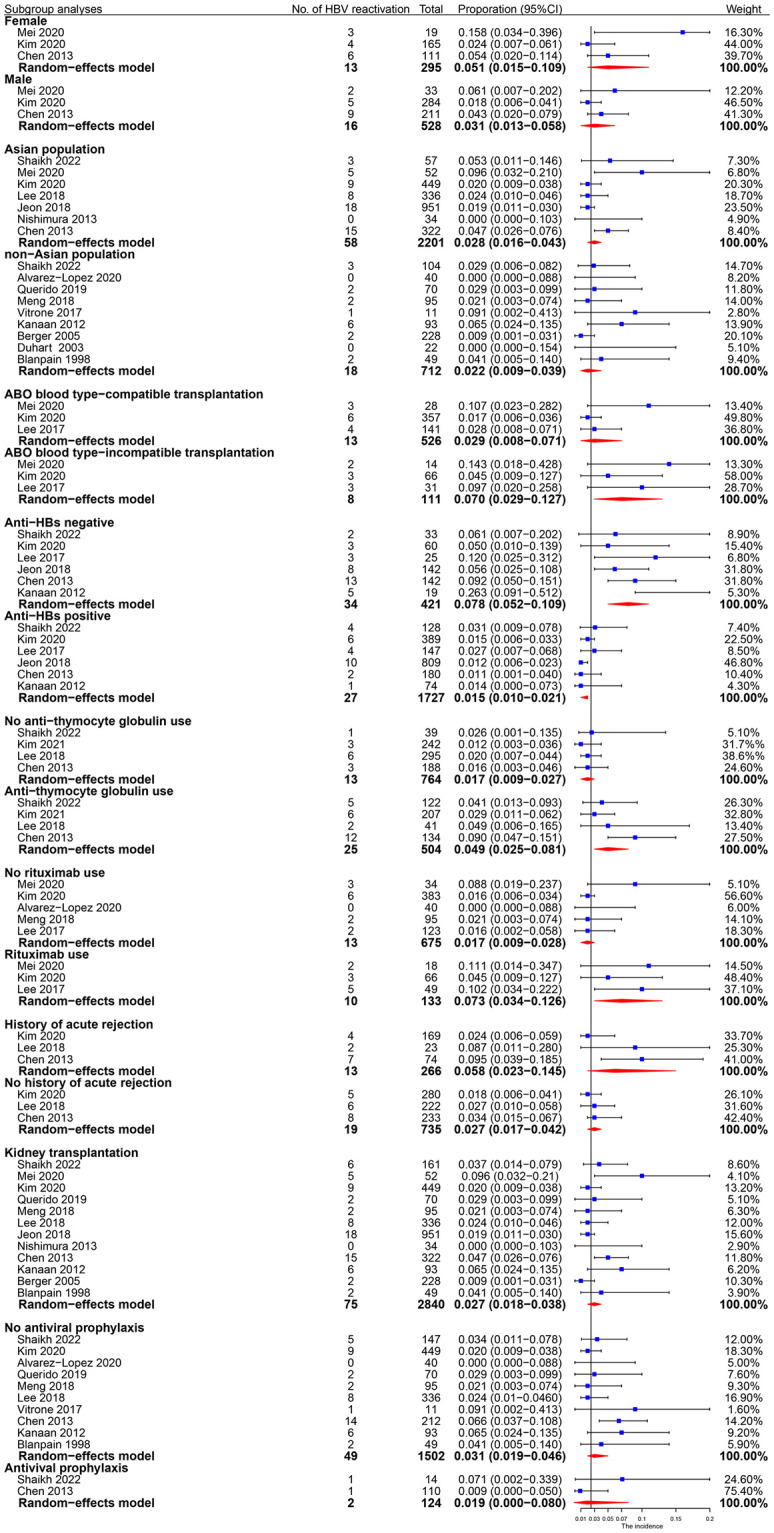
Incidence of HBV reactivation according to different patient characteristics. 95% CI, 95% confidence interval; anti-HBs, antibody against hepatitis b surface antigen; HBV, hepatitis b virus.

Further subgroup analyses were conducted. The incidence was 13/295 (random-effects, 5.1%; 95% CI [1.5%, 10.9%]; I^2^ = 60.7%) in female transplant recipients, and 16/528 (random-effects, 3.1%; 95% CI [1.3%, 5.8%]; I^2^ = 44.2%) in male recipients (Fig 3 and S4 Table); 58/2,201 (random-effects, 2.8%; 95% CI [1.6%, 4.3%]; I^2^ = 63.2%) in Asia, and 18/712 (random-effects, 2.2%; 95% CI [0.9%, 3.9%]; I^2^ = 44.9%) elsewhere; 13/526 (random-effects, 2.9%; 95% CI [0.8%, 7.1%]; I^2^ = 56.0%) in ABO blood type-compatible recipients, and 8/111 (random-effects, 7.0%; 95% CI [2.9%, 12.7%]; I^2^ = 0%) in ABO blood type-incompatible recipients; 13/764 (random-effects, 1.7%; 95% CI [0.9%, 2.7%]; I^2^ = 0%) in recipients not receiving ATG, and 25/504 (random-effects, 4.9%; 95% CI [2.5%, 8.1%]; I^2^ = 49.0%) in recipients receiving ATG; 34/421 (random-effects, 7.8%; 95% CI [5.2%, 10.9%]; I^2^ = 36.0%) in anti-HBs-negative recipients, and 27/1,727 (random-effects, 1.5%; 95% CI [1.0%, 2.1%]; I^2^ = 0%) in anti-HBs-positive recipients; 10/133 (random-effects, 7.3%; 95% CI [3.4%, 12.6%]; I^2^ = 0%) in recipients receiving rituximab, and 13/675 (random-effects, 1.7%; 95% CI [0.9%, 2.8%]; I^2^ = 41.5%) in recipients not receiving rituximab; 13/266 (random-effects, 5.8%; 95% CI [2.3%, 14.5%]; I^2^ = 63.2%) in recipients who experienced acute rejection, and 19/735 (random-effects, 2.7%; 95% CI [1.7%, 4.2%]; I^2^ = 0%) in recipients who did not experience acute rejection. Among 2,840 kidney transplant recipients, 65 (random-effects, 2.7%; 95% CI [1.8%, 3.8%]; I^2^ = 53.8%) experienced HBV reactivation. Among 1,502 recipients who did not receive any posttransplant antiviral prophylaxis, 49 (random-effects, 3.1%; 95% CI [1.9%, 4.6%]; I^2^ = 48.0%) experienced HBV reactivation. Among 124 recipients receiving antiviral prophylaxis, 2 experienced HBV reactivation (1.9%; 95% CI [0.0%, 8.0%]; I^2^ = 34.3%) experienced HBV reactivation.

### HBV-related complications caused by HBV reactivation

**Fig 4:**
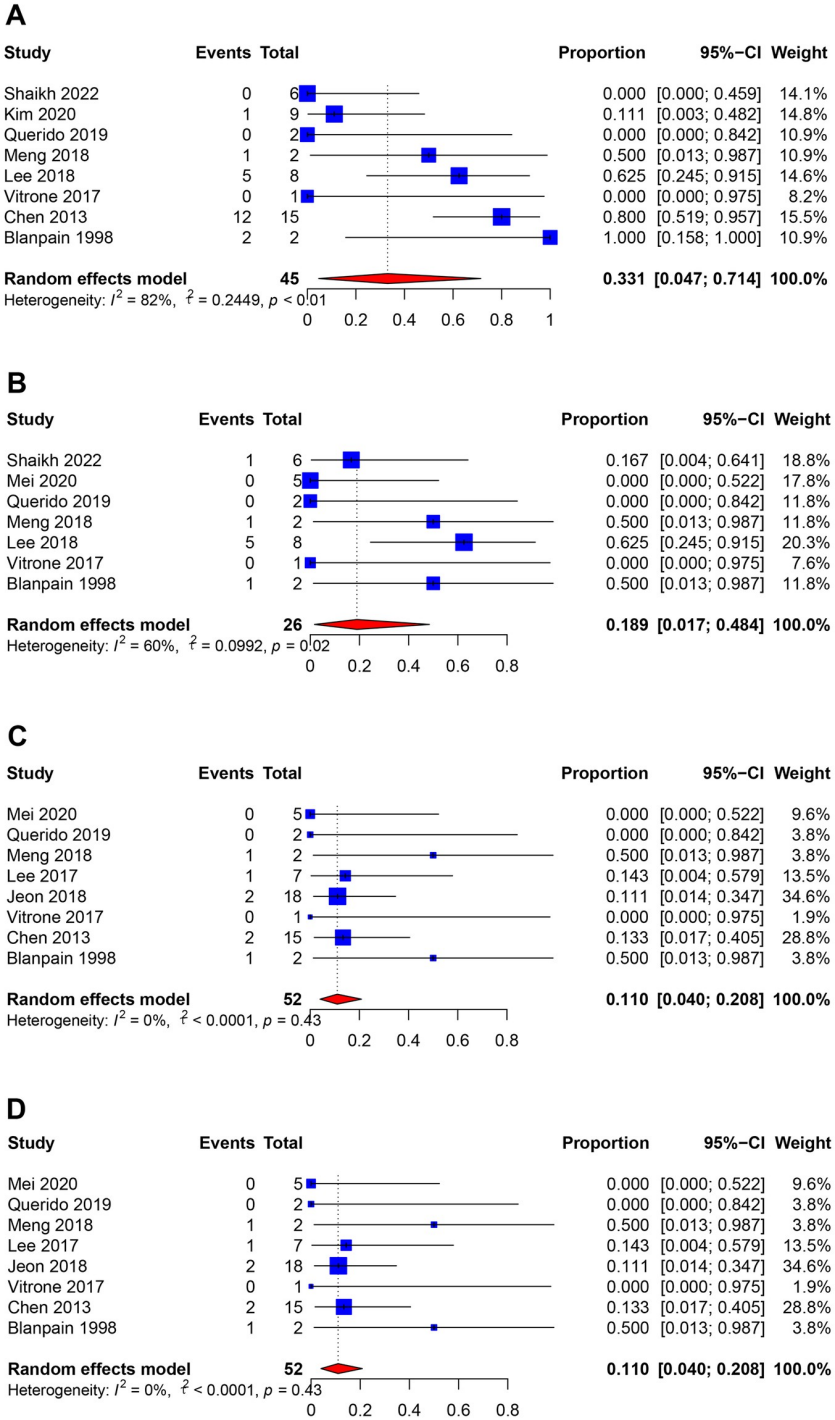
HBV-related complications after HBV reactivation. (A) Impaired liver function; (B) liver cirrhosis; (C) HBV-related hepatic failure; (D) HBV-related death. HBV, hepatitis b virus.

Eight studies reported that 21 of 45 recipients (random-effects, 33.1%; 95% CI [4.7%, 71.4%]; I^2^ = 82.4%) had impaired liver function after HBV reactivation (Fig 4A and S6 Table). Seven studies reported that 8 of 26 recipients (random-effects, 18.9%; 95% CI [1.7%, 48.4%]; I^2^ = 59.7%) developed hepatic cirrhosis after reactivation (Fig 4B). Eight studies reported 7 of 52 recipients (random-effects, 11.0%; 95% CI [4.0%, 20.8%]; I^2^ = 0.3%) developed HBV-related hepatic failure after HBV reactivation (Fig 4C). Eight studies reported 7 of 52 recipients experienced an HBV-related death (random-effects, 11.0%; 95% CI [4.0%, 20.8%]; I^2^ = 0.3%) after HBV reactivation (Table 2 and Fig 4D).

### Risk factors of HBV reactivation

**Fig 5:**
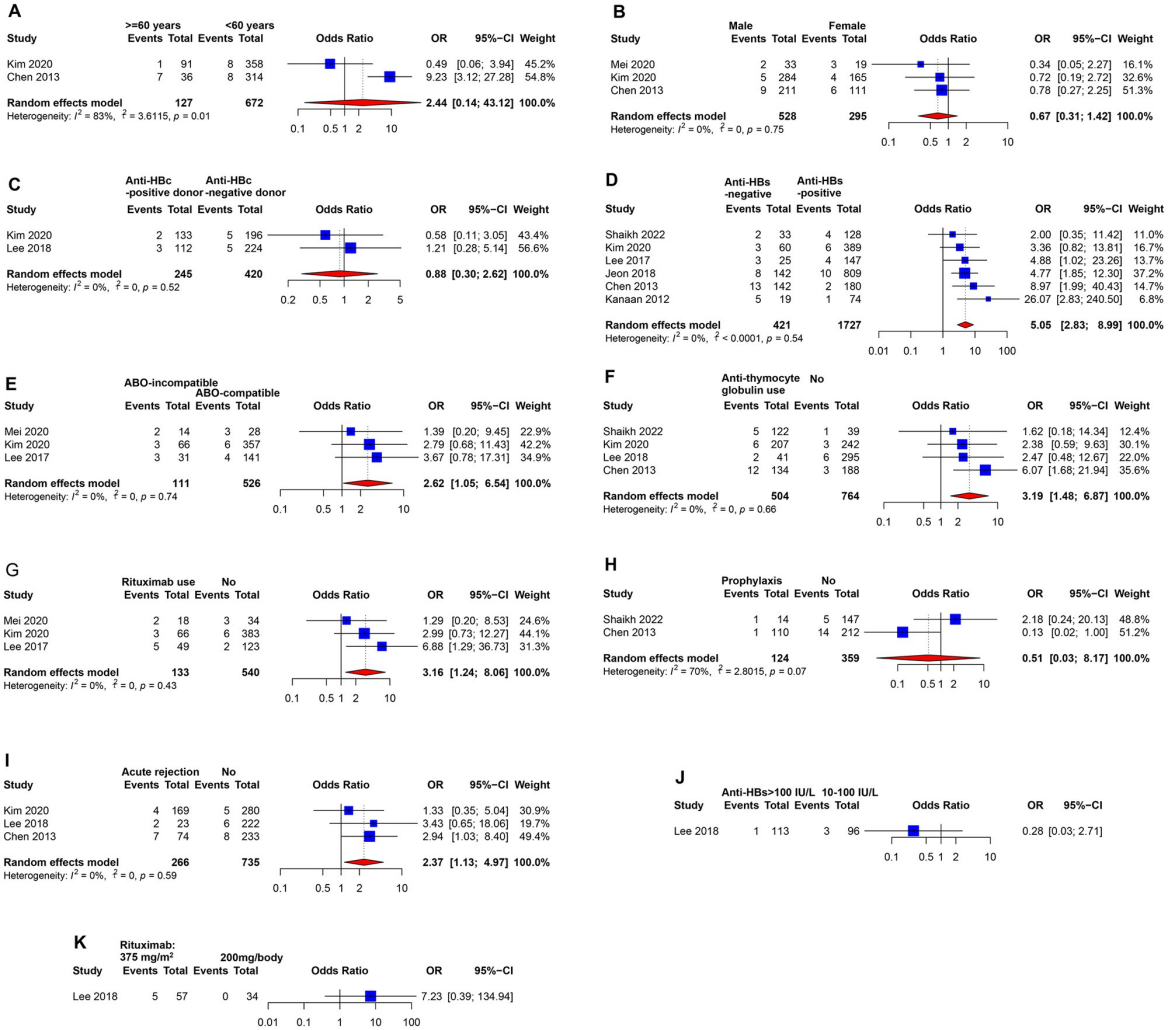
Forest plot of crude risk factors of HBV reactivation. Anti-HBc, antibody against hepatitis b core antigen; anti-HBs, antibody against hepatitis b surface antigen; OR, odds ratio; 95% CI, 95% confidence interval. (A) Age; (B) sex; (C) donors’ anti-HBc status; (D) recipients’ anti-HBs status; (E) ABO blood type-incompatible/compatible transplantation; (F) anti-thymocyte globulin use; (G) Rituximab use; (H) antiviral prophylaxis; (I) acute rejection history; (J) recipients’ anti-HBs levels: 100 IU/L vs. 10–100 IU/L; (K) rituximab dose: 375 mg/m^2^ vs. 200 mg/body. HBV, hepatitis b virus.

Nine studies reported crude risk factors of HBV reactivation (S3 Table). Pooled results showed that negative anti-HBs status (random-effects, 34/421 versus 27/1,727; crude odds ratio [OR] 5.05; 95% CI [2.83, 9.00]; *p* < 0.001) was associated with higher risk of HBV reactivation, without heterogeneity (I^2^ = 0%; p _heterogeneity_ = 0.539) (Fig 5 and S7 Table). Further analyses found that there was no statistically significant difference in the incidence of HBV reactivation in recipients with anti-HBs >100 IU/L compared with those with anti-HBs at 10 to 100 IU/L (random-effects, 1/113 versus 3/96, crude OR 0.28; 95% CI [0.03, 2.71]; *p* = 0.270). ABO blood type-incompatible transplantation was associated with an increased risk of HBV reactivation (random-effects, 8/111 versus 13/526; crude OR 2.62; 95% CI [1.05, 6.54]; *p* = 0.040), without heterogeneity (I^2^ = 0%; p _heterogeneity_ = 0.737). Acute rejection was associated with an increased risk of HBV reactivation (random-effects, 13/266 versus 19/735, crude OR 2.37; 95% CI [1.13, 4.97]; *p* = 0.022), without heterogeneity (I^2^ = 0%; p _heterogeneity_ = 0.586). Additionally, use of rituximab was associated with an increased risk (10/133 versus 11/540; crude OR 3.16; 95% CI [1.24, 8.06]; *p* = 0.016), without heterogeneity (I^2^ = 0%; p _heterogeneity_ = 0.429). Only 1 study explored the impact of rituximab dose. It showed a higher incidence of HBV reactivation in the standard-dose group (375 mg/m^2^) than in reduced-dose group (random-effects, 5/57 versus 0/34; crude OR 7.23; 95% CI [0.39, 134.94]), but this difference was not statistically significant (*p* = 0.185). Use of ATG was associated with a significantly higher incidence of HBV reactivation (random-effects, 25/504 versus 13/764; crude OR 3.19; 95% CI [1.48, 6.87]; *p* = 0.003), without heterogeneity (I^2^ = 0%; p _heterogeneity_ = 0.661). Two studies explored the impact of routine prophylaxis and found that routine prophylaxis was associated with a lower incidence of HBV reactivation (random-effects, 2/124 versus 19/359; crude OR 0.51; 95% CI [0.03, 8.17]), but this difference was not statistically significant (*p* = 0.637). Older age (*p* = 0.580), sex (*p* = 0.294), and positive anti-HBc (*p* = 0.82) in the donors were not associated with HBV reactivation.

**Fig 6:**
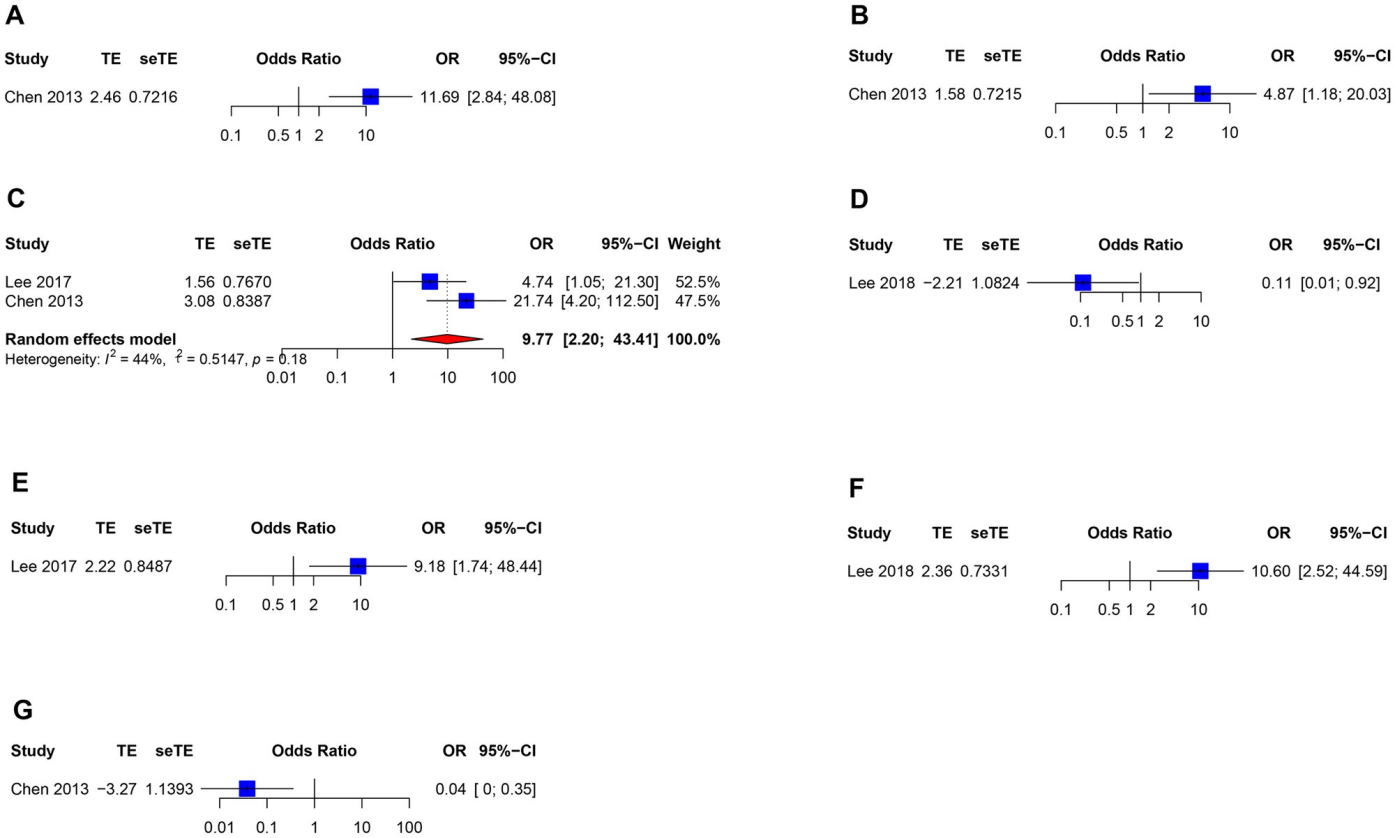
Forest plot of adjusted risk factors of HBV reactivation. Anti-HBs, antibody against hepatitis b surface antigen; OR, odds ratio; 95% CI, 95% confidence interval. (A) Age; (B) anti-thymocyte globulin use; (C) recipients’ anti-HBs status; (D) recipients’ anti-HBs levels: 100 IU/L vs. 10–100 IU/L; (E) rituximab use; (F) rituximab dose: 375 mg/m^2^ vs. 200 mg/body; (G) antiviral prophylaxis. HBV, hepatitis b virus.

However, only 3 studies reported adjusted risk factors of HBV reactivation (S3 Table). Pooled results were shown in Fig 6, showing ATG use (OR 4.87; 95% CI [1.18, 20.03]), anti-HBs negative status (OR 9.77; 95% CI [2.20, 43.41]), and anti-HBs negative status (OR 9.18; 95% CI [1.74, 48.44]) were associated with higher risk of HBV reactivation. However, antiviral prophylaxis contributed to lower risk of HBV reactivation (OR 0.04; 95% CI [0.10, 0.35]).

## Discussion

To our knowledge, this is the first systematic review and meta-analysis to determine the incidence, risk factors, and impact of HBV reactivation in non-liver SOT recipients with resolved HBV infection. Overall, the study showed a low incidence of HBV reactivation. However, once reactivated, there was a high risk of HBV-related hepatic failure and death. Hence, it is necessary to identify potential risk factors to identify those at a high risk of HBV reactivation. Our analyses identified negative anti-HBs status, ABO blood type-incompatible transplantation, ATG use, rituximab use, and a history of acute rejection as potential risk factors for HBV reactivation, indicating that, contrary to current guidelines, some non-liver SOT recipients with resolved HBV infection may benefit from routine monitoring and antiviral prophylaxis. Close monitoring and antiviral prophylaxis should be administered to patients at a high risk of HBV reactivation.

Based on the AGA and APASL guidelines, among patients with resolved HBV infection, immunosuppressants are categorized as high-risk (rituximab), moderate-risk (bortezomib), and low-risk group (immune checkpoint inhibitors) if the anticipated incidence of HBV reactivation is >10%, 1% to 10%, and <1%, respectively [13]. In patients in the moderate- and high-risk groups, the AGA recommends antiviral prophylaxis (strong recommendation for high-risk group; weak recommendation for moderate-risk group), to continue for at least 6 months after discontinuation of immunosuppressive therapy (at least 12 months for rituximab). In the field of transplantation, advanced immunosuppression (including rituximab, ATG, and bortezomib) has been widely used as a desensitization therapy for HLA-incompatible and ABO blood type-incompatible transplantation, treatment of antibody-mediated rejection, and induction therapy. However, the latest 2020 KDIGO Clinical Practice Guideline on the Evaluation and Management of Candidates for Kidney Transplantation recommends no antiviral prophylaxis in all patients with resolved HBV infection after transplantation [9].

Immunosuppressed patients with resolved HBV infection are at increased risk of HBV reactivation, and may also have more serious HBV-related complications once reactivated. In our analyses, the reported HBV reactivation rates varied considerably among studies, ranging from 0% to 10%. This difference resulted from the imbalanced baseline characteristics, different types of immunosuppression used, different antiviral prophylaxis regimens, and HBV immunity. Despite the low overall incidence, HBV reactivation is a cause for concern because HBV-related hepatic failure and HBV-related death occurred in more than 10% patients after HBV reactivation. These results highlight the importance of early detection and treatment of HBV reactivation, and underscore the importance of determining potential risk factors, identifying high-risk groups, and implementing early prevention.

Non-liver SOT recipients who were negative for anti-HBs had a higher risk of HBV reactivation. Similar results have been reported in patients resolved HBV infection receiving chemotherapy for hematologic malignancies [34,35]. In a recent meta-analysis including patients with hematologic malignancies and resolved HBV infection, Paul and colleagues [34] reported that the reactivation risk was 14% (95% CI [9.4%, 19%]) in 388 anti-HBs-negative patients versus 5.0% (95% CI [3.0%, 7.0%]) in 1,284 anti-HBs-positive patients (OR 0.21; 95% CI [0.14, 0.32]). Therefore, boosting HBV immunity by HBV vaccination might be beneficial in non-liver SOT recipients with resolved HBV infection. However, few studies have explored the role of HBV vaccination in this population. In a cohort of 46 patients who received allogeneic bone marrow transplantation [35], 21 received a standard 3-dose HBV vaccine posttransplant. Although HBV vaccination only resulted in the development of detectable anti-HBs antibodies in 9 patients, none of the 21 vaccinated patients experienced HBV reactivation compared with 12 of 25 unvaccinated patients after a median follow-up period of 60 months (*p* < 0.001). This suggests that HBV vaccination can prevent HBV activation even in patients who did not seroconvert to anti-HBs positivity.

Despite the presence of anti-HBs antibodies, the risk of reactivation was not completely eliminated. In our study, rituximab increased the risk of HBV reactivation. Standard-dose rituximab (375 mg/m^2^) has mainly been used to treat non-Hodgkin’s lymphoma, leukemia, and autoimmune disease. In a recent meta-analysis of 20 studies including 1,312 anti-HBc-positive lymphoma patients treated with rituximab-containing chemotherapy, the HBV reactivation rate was 9.0% (95% CI [5%, 15%]) [36]. Based on the AGA guidelines, antiviral prophylaxis for 12 months after discontinuation of rituximab is strongly recommended [13]. Notably, in the field of non-liver SOT, reduced-dose rituximab (100 to 300 mg/body) is used to eliminate lymphocyte B cells in ABO blood type-incompatible transplant recipients and was associated with a lower incidence of HBV reactivation than with standard-dose rituximab [26]. In addition to rituximab, plasmapheresis is commonly used to deplete anti-donor blood-type antibodies in ABO blood type-incompatible transplant recipients. Notably, theoretically, plasmapheresis can also remove anti-HBs. This may explain why ABO blood type-incompatible transplantation is associated with a higher risk of HBV reactivation. Based on the AGA and EASL recommendations [6,13], antiviral prophylaxis should be considered in these patients.

Additionally, our study also showed that the use of the T-lymphocyte-depleting agent, ATG, was associated with a higher risk of HBV reactivation. The American Society of Transplantation recommends lymphocyte-depleting agents as induction therapy for recipients with high immunological risk (Grade: 2B) [37]. In addition, rituximab, high-dose steroids, and ATG are commonly used to treat antibody-mediated or T-lymphocyte-mediated, **acute rejection**. Our results also show that patients experiencing **acute rejection** had a higher risk of HBV reactivation. Hence, antiviral prophylaxis should be considered for these recipients.

In this meta-analysis, only 2 studies explored the impact of routine antiviral prophylaxis [14,33]. In a retrospective analysis by Chen and colleagues [14], 110 kidney transplant recipients received lamivudine 100 mg daily for 3 months. Only 1 developed HBV reactivation compared with 14 of 212 patients without prophylaxis (adjusted OR: 0.038; 95% CI [0.004, 0.348]; *p* = 0.004). Notably, the role of antiviral prophylaxis in preventing HBV reactivation has been well demonstrated in other immune system diseases. In a randomized clinical trial by Huang and colleagues [38], 80 patients with lymphoma and resolved HBV infection were randomly assigned to receive either prophylactic entecavir before chemotherapy to 3 months after completing chemotherapy (*n* = 41) or therapeutic entecavir at the time of HBV reactivation (*n* = 39). During a mean 18-month follow-up period, 1 patient (2.4%) in the prophylactic group and 7 patients (17.9%) in the control group developed HBV reactivation (*p* = 0.027). The cumulative HBV reactivation rates were 8%, 11.2%, and 25.9% in the therapeutic group and 0%, 0%, and 4.3% in the prophylactic group (*p* = 0.019) at 6, 12, and 18 months after chemotherapy, respectively.

This meta-analysis had several limitations. First, there was moderate heterogeneity in the incidence of HBV reactivation. However, such heterogeneity is not surprising, given the differences in baseline characteristics, immunosuppression regimens, HBV immunity, and posttransplant antiviral prophylaxis. Despite these differences, the overall risk of HBV reactivation was not negligible in non-liver SOT recipients with resolved HBV infection. Subgroup analyses identified recipients at higher risk. Second, despite the inclusion of all non-liver SOT studies, most studies were conducted in kidney transplant recipients. Thus, these results are primarily applicable to kidney transplant recipients and further research is required on the risk of HBV reactivation in other non-liver SOT recipients. Third, most of the included studies did not report adjusted estimates of risk factors. Future studies were still wanted to confirm the current findings. Fourth, despite our attempts to explore the benefits of antiviral prophylaxis, only 2 studies reported on this. Based on our results, further studies should investigate the effect of antiviral prophylaxis and vaccination in high-risk populations, and also assess the need for HBV serology monitoring in low-risk populations. Last, of the 16 studies included in the meta-analysis, 7 were conducted in East Asia and 9 were conducted in Europe and America. According to the World Health Organization Global Hepatitis Report 2017, HBV prevalence (positive HBsAg) has geographical variation with 6.0% to 7.0% in Africa and East Asia but 0.5% to 2.0% in Europe and America [1]. This geographic variation should be considered in future research.

In conclusion, this systematic review and meta-analysis examined the incidence of HBV reactivation in non-liver SOT recipients with resolved HBV infection. Despite low overall incidence, the incidence differed according to specific characteristics. Once HBV reactivation occurred, there was a high risk of HBV-related hepatic failure and death. We also identified several risk factors for HBV reactivation, including patients without HBV immunity, patients receiving ATG and rituximab therapy, and patients receiving ABO blood type-incompatible transplantation, or experiencing acute rejection. Close monitoring and antiviral prophylaxis should be considered in these patients. Further research should investigate the efficacy and safety of antiviral prophylaxis in high-risk populations.

## Supporting information

S1 Table Search strategy. (DOCX)

S2 Table Result of the Newcastle–Ottawa scale quality assessment. (DOCX)

S3 Table Definition of resolved hepatitis b virus infection, hepatitis b virus reactivation, and hepatitis b virus-related complications. (DOCX)

S4 Table Incidence of hepatitis b virus reactivation in non-liver solid organ transplant recipients. (DOCX)

S5 Table Results of meta-regression analyses. (DOCX)

S6 Table Meta-analyses of hepatitis b virus-related complications. (DOCX)

S7 Table Risk factors of hepatitis b virus reactivation in non-liver solid organ transplant recipients with resolved hepatitis b virus infection. (DOCX)

S1 Fig Funnel plot and sensitivity analysis of the risk of hepatitis b virus reactivation. (PDF)

S1 PRISMA Checklist PRISMA 2020 Checklist. (DOCX)

S1 Data Data and R code underlying the current findings. (ZIP)

## Abbreviations

AGA: American Gastroenterological Association
anti-HBc: antibody against hepatitis b core antigen
anti-HBs: antibody against hepatitis b surface antigen
APASL: Asian-Pacific Association for the Study of the Liver
ATG: anti-thymocyte immunoglobulin
ccc DNA: closed circular DNA
CI: confidence interval
EASL: European Association for the Study of the Liver
HBeAg: hepatitis b e antigen
HBsAg: hepatitis b surface antigen
HBV: hepatitis b virus
HCV: hepatitis c virus
HLA: human leukocyte antigen
KDIGO: Kidney Diseases Improving Global Outcomes
KT: kidney transplantation
LT: liver transplantation
NOS: Newcastle–Ottawa scale
OR: odds ratio
SOT: solid organ transplantation
