# Supplementary material for: Incidence, risk factors, and clinical outcomes of HBV reactivation in non-liver solid organ transplant recipients with resolved HBV infection: A systematic review and meta-analysis
Source: PLoS Med. 2023 Mar 15;20(3):e1004196. doi: 10.1371/journal.pmed.1004196 (PMC10058170; doi:10.1371/journal.pmed.1004196)
Supplement: S1 Table — (DOCX) [file pmed.1004196.s001.docx]

S1-table : Search Strategy

| Embase search strategy |  |
| --- | --- |
| 1 | exp hepatitis B virus/or exp hepatitis B/ |
| 2 | exp hepatitis B surface antigen/ |
| 3 | exp hepatitis B surface antibody/ |
| 4 | exp hepatitis B core antibody/ |
| 5 | exp "hepatitis B(e) antigen"/ |
| 6 | exp "hepatitis B(e) antibody"/ |
| 7 | exp kidney transplantation/ |
| 8 | exp kidney graft/ |
| 9 | exp heart transplantation/ |
| 10 | exp lung transplantation/ |
| 11 | exp pancreas transplantation/ |
| 12 | 1 or 2 or 3 or 4 or 5 or 6 |
| 13 | 7 or 8 or 9 or 10 or 11 |
| 14 | 12 and 13 |
