# Supplementary material for: Incidence, risk factors, and clinical outcomes of HBV reactivation in non-liver solid organ transplant recipients with resolved HBV infection: A systematic review and meta-analysis
Source: PLoS Med. 2023 Mar 15;20(3):e1004196. doi: 10.1371/journal.pmed.1004196 (PMC10058170; doi:10.1371/journal.pmed.1004196)
Supplement: S2 Table — (DOCX) [file pmed.1004196.s002.docx]

S2 Table: Result of the Newcastle-Ottawa scale quality assessment.

| Author Year | Total NOS score | Selection (4 score) | | | | Comparability (2 score) | Outcome (3 score) | | |
| --- | --- | --- | --- | --- | --- | --- | --- | --- | --- |
|  |  | Representativeness of the exposed cohort (1 score) | Selection of the non-exposed cohort (1 score) | Ascertainment of exposure (1 score) | Outcomes were not present at study initiation (1 score) | Comparability of cohorts on the basis of the design or analysis | Assessment of outcome of follow-up (1 score) | Was follow-up long enough for outcome to occur (1 score) | Adequacy (1 score) |
| Shaikh 2022^[1]^ | 6 | 1 | 1 | 1 | 1 | 0 | 1 | 1 | 0 |
| Mei 2020^[2]^ | 5 | 1 | 0 | 1 | 1 | 0 | 1 | 1 | 0 |
| Kim 2020^[3]^ | 7 | 1 | 1 | 1 | 1 | 1 | 1 | 1 | 0 |
| Alvarez-Lopez 2020^[4]^ | 7 | 1 | 1 | 1 | 1 | 2 | 1 | 0 | 0 |
| Querido 2019^[5]^ | 6 | 1 | 0 | 1 | 1 | 1 | 1 | 1 | 0 |
| Meng 2018^[6]^ | 7 | 1 | 0 | 1 | 1 | 2 | 1 | 1 | 0 |
| Lee 2018^[7]^ | 7 | 1 | 1 | 1 | 1 | 1 | 1 | 1 | 0 |
| Jeon 2018^[8]^ | 8 | 1 | 1 | 1 | 1 | 1 | 1 | 1 | 1 |
| Vitrone 2017^[9]^ | 5 | 1 | 0 | 1 | 1 | 0 | 1 | 1 | 0 |
| Lee 2017^[10]^ | 7 | 1 | 0 | 1 | 1 | 1 | 1 | 1 | 1 |
| Nishimura 2013^[11]^ | 4 | 0 | 1 | 1 | 1 | 0 | 0 | 1 | 0 |
| Chen 2013^[12]^ | 6 | 1 | 0 | 1 | 1 | 1 | 1 | 1 | 0 |
| Kanaan 2012^[13]^ | 5 | 1 | 0 | 1 | 1 | 1 | 0 | 1 | 0 |
| Berger 2005^[14]^ | 4 | 1 | 0 | 1 | 1 | 1 | 0 | 0 | 0 |
| Duhart 2003^[15]^ | 5 | 1 | 0 | 1 | 1 | 0 | 1 | 1 | 0 |
| Blanpain 1998^[16]^ | 6 | 1 | 0 | 1 | 1 | 1 | 1 | 0 | 1 |

NOS: Newcastle-Ottawa scale

**Reference:**

1. Shaikh SA, Kahn J, Aksentijevic A, Kawewat-Ho P, Bixby A, Rendulic T, et al. A multicenter evaluation of hepatitis B reactivation with and without antiviral prophylaxis after kidney transplantation. Transpl Infect Dis. 2022 Feb;24(1):e13751. doi: 10.1111/tid.13751. Epub 2021 Dec 7. PMID: 34725887.
2. Mei T, Noguchi H, Hisadome Y, Kaku K, Nishiki T, Okabe Y, et al. Hepatitis B virus reactivation in kidney transplant patients with resolved hepatitis B virus infection: Risk factors and the safety and efficacy of preemptive therapy. Transpl Infect Dis. 2020 Apr;22(2):e13234. doi: 10.1111/tid.13234. Epub 2020 Feb 6. PMID: 31856328.
3. Kim J, Chung SJ, Sinn DH, Lee KW, Park JB, Huh W, et al. Hepatitis B reactivation after kidney transplantation in hepatitis B surface antigen-negative, core antibody-positive recipients. J Viral Hepat. 2020 Jul;27(7):739-746. doi: 10.1111/jvh.13279. Epub 2020 Feb 28. PMID: 32057171.
4. Álvarez-López P, Riveiro-Barciela M, Oleas-Vega D, Flores-Cortes C, Román A, Perelló M, et al. Anti-HBc impacts on the risk of hepatitis B reactivation but not on survival of solid-organ transplant recipients. Medicine (Baltimore). 2020 Feb;99(9):e19407. doi: 10.1097/MD.0000000000019407. PMID: 32118794
5. Querido S, Weigert A, Adragão T, Rodrigues L, Jorge C, Bruges M, et al. Risk of hepatitis B reactivation in hepatitis B surface antigen seronegative and core antibody seropositive kidney transplant recipients. Transpl Infect Dis. 2019 Feb;21(1):e13009. doi: 10.1111/tid.13009. Epub 2018 Nov 5. PMID: 30295412.
6. Meng C, Belino C, Pereira L, Pinho A, Sampaio S, Tavares I, et al. Reactivation of Hepatitis B virus in kidney transplant recipients with previous clinically resolved infection: A single-center experience. Nefrologia (Engl Ed). 2018 Sep-Oct;38(5):545-550. doi: 10.1016/j.nefro.2018.02.004. Epub 2018 Apr 27. PMID: 29709320.
7. Lee J, Park JY, Kim DG, Lee JY, Kim BS, Kim MS, et al. Effects of rituximab dose on hepatitis B reactivation in patients with resolved infection undergoing immunologic incompatible kidney transplantation. Sci Rep. 2018 Oct 23;8(1):15629. doi: 10.1038/s41598-018-34111-5. PMID: 30353021
8. Jeon JW, Kim SM, Cho H, Baek CH, Kim H, Shin S, et al. Presence of Hepatitis B Surface Antibody in Addition to Hepatitis B Core Antibody Confers Protection Against Hepatitis B Virus Infection in Hepatitis B Surface Antigen-negative Patients Undergoing Kidney Transplantation. Transplantation. 2018 Oct;102(10):1717-1723. doi: 10.1097/TP.0000000000002173. PMID: 29621059.
9. Vitrone M, Iossa D, Rinaldi L, Pafundi PC, Molaro R, Parrella A, et al. Hepatitis B virus reactivation after heart transplant: Incidence and clinical impact. J Clin Virol. 2017 Nov;96:54-59. doi: 10.1016/j.jcv.2017.09.011. Epub 2017 Sep 23. PMID: 28964958.
10. Lee J, Park JY, Huh KH, Kim BS, Kim MS, Kim SI, et al. Rituximab and hepatitis B reactivation in HBsAg-negative/anti-HBc-positive kidney transplant recipients. Nephrol Dial Transplant. 2017 May 1;32(5):906. doi: 10.1093/ndt/gfx048. PMID: 28371939.
11. Nishimura K, Kishikawa H, Yoshida Y, Ueda N, Nakazawa S, Yamanaka K, et al. Clinical and virologic courses of hepatitis B surface antigen-negative and hepatitis B core or hepatitis B surface antibody-positive renal transplant recipients. Transplant Proc. 2013 May;45(4):1600-2. doi: 10.1016/j.transproceed.2013.01.093. PMID: 23726628.
12. Chen GD, Gu JL, Qiu J, Chen LZ. Outcomes and risk factors for hepatitis B virus (HBV) reactivation after kidney transplantation in occult HBV carriers. Transpl Infect Dis. 2013 Jun;15(3):300-5. doi: 10.1111/tid.12065. Epub 2013 Mar 8. PMID: 23473005.
13. Rücker G, Schwarzer G, Carpenter J, Olkin I. Why add anything to nothing? The arcsine difference as a measure of treatment effect in meta-analysis with zero cells. Stat Med. 2009 Feb 28;28(5):721-38. doi: 10.1002/sim.3511. PMID: 19072749.
14. Berger A, Preiser W, Kachel HG, Stürmer M, Doerr HW. HBV reactivation after kidney transplantation. J Clin Virol. 2005 Feb;32(2):162-5. doi: 10.1016/j.jcv.2004.10.006. PMID: 15653420.
15. Duhart BT Jr, Honaker MR, Shokouh-Amiri MH, Riely CA, Vera SR, Taylor SL, et al. Retrospective evaluation of the risk of hepatitis B virus reactivation after transplantation. Transpl Infect Dis. 2003 Sep;5(3):126-31. doi: 10.1034/j.1399-3062.2003.00021.x. PMID: 14617300.
16. Blanpain C, Knoop C, Delforge ML, Antoine M, Peny MO, Liesnard C, Vereerstraeten P, Cogan E, Adler M, Abramowicz D. Reactivation of hepatitis B after transplantation in patients with pre-existing anti-hepatitis B surface antigen antibodies: report on three cases and review of the literature. Transplantation. 1998 Oct 15;66(7):883-6. doi: 10.1097/00007890-199810150-00012. PMID: 9798698.
