# Supplementary material for: Incidence, risk factors, and clinical outcomes of HBV reactivation in non-liver solid organ transplant recipients with resolved HBV infection: A systematic review and meta-analysis
Source: PLoS Med. 2023 Mar 15;20(3):e1004196. doi: 10.1371/journal.pmed.1004196 (PMC10058170; doi:10.1371/journal.pmed.1004196)
Supplement: S3 Table — (DOCX) [file pmed.1004196.s003.docx]

S3 Table: Definition of resolved hepatitis b virus infection, hepatitis b virus reactivation, and hepatitis b virus-related complications

| Author | Sample size | HBV reactivation | Resolved HBV infection definition | HBV reactivation definition | HBV serology monitoring | Crude risk factors of HBV reactivation | Adjusted risk factors of HBV reactivation | Definition of impaired liver function | Definition of liver cirrhosis | Definition of HBV-related hepatic failure | Treatment and prognosis of HBV reactivation |
| --- | --- | --- | --- | --- | --- | --- | --- | --- | --- | --- | --- |
| Shaikh 2022^[1]^ | 161 | 6 | HBsAg-negative and anti-HBc-positive | Positive HBV PCR of any viral loadat or above the minimal detection level | NA | Age, anti-HBs status, ATG, HBV prophylaxis | NA | Elevation in liver enzymes 2-3 fold of the normal limit | Metavir histological index of grading fibrosis | NA | NA |
| Mei 2020^[2]^ | 52 | 5 | Seronegativity for HBs Ag and seropositivity for anti-HBc and/or anti-HBs, without the presence of HBV DNA | Seropositivity for HBV DNA at or above the minimal detection level of 1.0 log IU/mL | HBsAg, anti-HBc, and anti-HBs were measured using commercial chemiluminescent immunoassay kits (Lumipulse PrestoII assay; Fujirebio, Inc); the quantitative PCR to monitor HBV DNA (COBAS 6800 system; Roche Molecular Diagnostics). | Age, Sex, Duration of dialysis, ABO blood type-incompatible transplantation, anti-HBc status, anti-HBs status, Rituximab | NA | NA | NA | NA | Three patients took entecavir; the other two underwent follow-up without medical treatment. Three patients who were treated became negative for serum HBV DNA after 1 or 2 months. Patients who were not treated maintained a serum HBV DNA level of <1.3 log IU/mL; one patient became negative for serum HBV-DNA after 1 month, whereas the other patient continued to exhibit serum HBV DNA for 11 months. |
| Kim 2020^[3]^ | 449 | 9 | HBsAg negative, HBV DNA negative, anti-HBc positive | HBsAg seroreversion | NA | Age, Sex, anti-HBs status, ABO blood type-incompatible transplantation, Rituximab, ATG, Plasmapheresis | NA | Increase in alanine aminotransferase levels above 5-fold the upper limit of the normal range. | NA | Liver failure was defined as an acute hepatic insult manifesting as jaundice (serum bilirubin ≥5 mg/dL and coagulopathy (international normalized ratio ≥1.5 or prothrombin activity <40%) complicated within 4 weeks by clinical ascites and/or encephalopathy | Entecavir, without reporting treatment course and clical outcomes |
| Alvarez-Lopez 2020^[4]^ | 40 | 0 | HBsAg negative, HBV DNA negative, anti-HBc positive | Rise in HBV DNA compared with baseline or reappearance of HBsAg | Serum HBVDNA was PCR quantified with a COBAS 6800 HBV system (Roche Diagnostics, Mannheim, Germany) | NA | NA | NA | NA | NA | NA |
| Querido 2019^[5]^ | 70 | 2 | HBsAg negative and anti-HBc positive, regardless of anti‐HBs status, in addition to liver enzymes in the normal range. | detection of HBV DNA viral load >2000 IU/mL | NA | NA | NA | NA | NA | NA | Patient 1: The time between transplantation and HBV reactivation was 41 months. He started antiviral therapy with entecavir and, after that, viral load started to decline but is still positive 6 months after the diagnosis. Liver enzymes always remain in the normal range; Patient 2: Entecavir, without reporting treatment course and clical outcomes |
| Meng 2018^[6]^ | 95 | 2 | Seropositivity for anti-HBc without detectable HBsAg, and normal liver enzymes (ALT, AST), with or without anti-HBs positive titers. | viral load >2000 IU/ml. | Serological markers were obtained using hemi luminescent methods (Prism HBsAg, and Prism HBcore , Abbott; Architect Anti-HBs , Abbott); and HBV DNA was obtained using multiplex nucleic acid test, COBAS Test, version 2.0, Roche | NA | NA | NA | NA | NA | Patient 1: The patient was started on entecavir, but expired a week later due to acute liver failure; Patient 2: Entecavir was started with disappearance of HBsAg and viremia, which became undetectable |
| Lee 2018^[7]^ | 336 | 8 | NA | NA | NA | Rituximab, anti-HBs status, ATG, Donor anti-HBc | Rituximab, anti-HB (adjusted by rejection, ATG use, donor anti-HBc status, causes of rituximab, number of plasmapheresis, tacrolimus trough level, MMF dose) | Hepatitis flare was defined as ≥3-fold increase in serum ALT levels that exceeded 100 IU/L. HBV-related hepatitis flare was defined as hepatitis flare with HBV reactivation, in the absence of laboratory features of acute infection with hepatitis A virus, HCV, or cytomegalovirus | NA | NA | All patients initiated entecavir upon detection of reactivation. One patient in the standard-dose rituximab group died of hepatic failure despite active antiviral treatment. Another patient in the standard-dose rituximab group died due to unknown causes 20 months after HBV reactivation. |
| Jeon 2018^[8]^ | 951 | 18 | HBsAg negative, HBV DNA negative, anti-HBc positive | appearance of HBsAg and/or HBV DNA. | Serum HBV DNA concentration was measured using a hybrid capture signal amplification assay (Digene Corporation, Gaithersburg, MD, USA) before January 2005; Beginning in January 2005, HBV DNA level was measured using real-time polymerase chain reaction (Abbott Laboratories) | Anti-HBs status | NA | NA | liver cirrhosis on the CT suggestive of alcoholic cirrhosis | Liver failure was defined as the development of severe liver injury with encephalopathy and impaired synthetic function (international normalized ratio of prothrombin time of ≥1.5). | NA |
| Vitrone 2017^[9]^ | 11 | 1 | HBsAg negative, HBV DNA negative, anti-HBc positive | NA | HBV by commercial real-time PCR (Cobas TaqMan HBVDNA, Roche Diagnostics). | NA | NA | NA | NA | NA | Antiviral treatment with entecavir, 0.5 mg once daily, was started and is currently ongoing. |
| Lee 2017^[10]^ | 172 | 7 | HBsAg-negative and anti-HBc-positive, without HBV DNA | the reappearance of hepatitis B surface antigen or HBV DNA. | Serum HBsAg, anti-HBc and anti-HBs were evaluated using commercially available enzyme immunoassays (Abbott Diagnostics, Abbott Park, IL, USA). | ATG, Rituximab, Anti-HBs, donor anti-HBc | Rituximab, anti-HBs (adjusted by rejection, ATG use, donor anti-HBc status) | Severe hepatitis was defined as more than a 10-fold increase of serum ALT above the upper limit of normal or more than a 2-fold increase of bilirubin above the ULN. HBV-related severe hepatitis was defined as severe hepatitis with HBV reactivation, in the absence of laboratory features of acute infection with hepatitis A virus, HCV or cytomegalovirus. | NA | NA | NA |
| Nishimura 2013^[11]^ | 34 | 0 | HBsAg negative, HBV DNA negative, anti-HBc positive | NA | NA | NA | NA | NA | NA | NA | NA |
| Chen 2013^[12]^ | 322 | 15 | HBsAg negative, HBV DNA negative, anti-HBc positive | NA | NA | Age, anti-HBs status, prophylaxis, ATG, sex | Age, ATG, anti-HBs, prophylaxis (adjusted by sex, dialysis time, delayed graft function, tacrolimus, MMF) | NA | NA | NA | Lamivudine 100 mg daily was given when HBV reactivation or reinfection was diagnosed. If lamivudine could not control HBV infection, adefovir or entecavir were used as substitute antivirus drugs |
| Kanaan 2012^[13]^ | 93 | 6 | HBsAg negative, anti-HBc positive, anti-HBs positive or negative, and normal liver enzymes | HBsAg reversion with HBV DNA > 2000 IU/mL. | HBV DNA was tested using the Abbott HBV RealTime assay (Abbott Laboratories, North Chicago, IL, USA) | anti-HBs status, donor type | NA | NA | NA | NA | NA |
| Berger 2005^[14]^ | 228 | 2 | NA | NA | NA | NA | NA | NA | NA | NA | NA |
| Duhart 2003^[15]^ | 22 | 0 | HBsAg negative, HBV DNA negative, anti-HBc positive | Appearance of serum HBsAg and/or HBV DNA | NA | NA | NA | NA | NA | NA | NA |
| Blanpain 1998^[16]^ | 49 | 2 | NA | NA | NA | NA | NA | NA | NA | NA | NA |

HBV: hepatitis b virus; HBsAg: hepatitis b surface antigen; anti-HBs: antibody against hepatitis b surface antigen; anti-HBc: antibody against hepatitis b core antigen; ATG: anti-thymocyte globulin; NA: not available; PCR: polymerase chain reaction; AST: aspartate amino transferase; ALT: alanine amino transferase, MMF: mycophenolate mofetil.

**Reference:**

1. Shaikh SA, Kahn J, Aksentijevic A, Kawewat-Ho P, Bixby A, Rendulic T, et al. A multicenter evaluation of hepatitis B reactivation with and without antiviral prophylaxis after kidney transplantation. Transpl Infect Dis. 2022 Feb;24(1):e13751. doi: 10.1111/tid.13751. Epub 2021 Dec 7. PMID: 34725887.
2. Mei T, Noguchi H, Hisadome Y, Kaku K, Nishiki T, Okabe Y, et al. Hepatitis B virus reactivation in kidney transplant patients with resolved hepatitis B virus infection: Risk factors and the safety and efficacy of preemptive therapy. Transpl Infect Dis. 2020 Apr;22(2):e13234. doi: 10.1111/tid.13234. Epub 2020 Feb 6. PMID: 31856328.
3. Kim J, Chung SJ, Sinn DH, Lee KW, Park JB, Huh W, et al. Hepatitis B reactivation after kidney transplantation in hepatitis B surface antigen-negative, core antibody-positive recipients. J Viral Hepat. 2020 Jul;27(7):739-746. doi: 10.1111/jvh.13279. Epub 2020 Feb 28. PMID: 32057171.
4. Álvarez-López P, Riveiro-Barciela M, Oleas-Vega D, Flores-Cortes C, Román A, Perelló M, et al. Anti-HBc impacts on the risk of hepatitis B reactivation but not on survival of solid-organ transplant recipients. Medicine (Baltimore). 2020 Feb;99(9):e19407. doi: 10.1097/MD.0000000000019407. PMID: 32118794
5. Querido S, Weigert A, Adragão T, Rodrigues L, Jorge C, Bruges M, et al. Risk of hepatitis B reactivation in hepatitis B surface antigen seronegative and core antibody seropositive kidney transplant recipients. Transpl Infect Dis. 2019 Feb;21(1):e13009. doi: 10.1111/tid.13009. Epub 2018 Nov 5. PMID: 30295412.
6. Meng C, Belino C, Pereira L, Pinho A, Sampaio S, Tavares I, et al. Reactivation of Hepatitis B virus in kidney transplant recipients with previous clinically resolved infection: A single-center experience. Nefrologia (Engl Ed). 2018 Sep-Oct;38(5):545-550. doi: 10.1016/j.nefro.2018.02.004. Epub 2018 Apr 27. PMID: 29709320.
7. Lee J, Park JY, Kim DG, Lee JY, Kim BS, Kim MS, et al. Effects of rituximab dose on hepatitis B reactivation in patients with resolved infection undergoing immunologic incompatible kidney transplantation. Sci Rep. 2018 Oct 23;8(1):15629. doi: 10.1038/s41598-018-34111-5. PMID: 30353021
8. Jeon JW, Kim SM, Cho H, Baek CH, Kim H, Shin S, et al. Presence of Hepatitis B Surface Antibody in Addition to Hepatitis B Core Antibody Confers Protection Against Hepatitis B Virus Infection in Hepatitis B Surface Antigen-negative Patients Undergoing Kidney Transplantation. Transplantation. 2018 Oct;102(10):1717-1723. doi: 10.1097/TP.0000000000002173. PMID: 29621059.
9. Vitrone M, Iossa D, Rinaldi L, Pafundi PC, Molaro R, Parrella A, et al. Hepatitis B virus reactivation after heart transplant: Incidence and clinical impact. J Clin Virol. 2017 Nov;96:54-59. doi: 10.1016/j.jcv.2017.09.011. Epub 2017 Sep 23. PMID: 28964958.
10. Lee J, Park JY, Huh KH, Kim BS, Kim MS, Kim SI, et al. Rituximab and hepatitis B reactivation in HBsAg-negative/anti-HBc-positive kidney transplant recipients. Nephrol Dial Transplant. 2017 May 1;32(5):906. doi: 10.1093/ndt/gfx048. PMID: 28371939.
11. Nishimura K, Kishikawa H, Yoshida Y, Ueda N, Nakazawa S, Yamanaka K, et al. Clinical and virologic courses of hepatitis B surface antigen-negative and hepatitis B core or hepatitis B surface antibody-positive renal transplant recipients. Transplant Proc. 2013 May;45(4):1600-2. doi: 10.1016/j.transproceed.2013.01.093. PMID: 23726628.
12. Chen GD, Gu JL, Qiu J, Chen LZ. Outcomes and risk factors for hepatitis B virus (HBV) reactivation after kidney transplantation in occult HBV carriers. Transpl Infect Dis. 2013 Jun;15(3):300-5. doi: 10.1111/tid.12065. Epub 2013 Mar 8. PMID: 23473005.
13. Rücker G, Schwarzer G, Carpenter J, Olkin I. Why add anything to nothing? The arcsine difference as a measure of treatment effect in meta-analysis with zero cells. Stat Med. 2009 Feb 28;28(5):721-38. doi: 10.1002/sim.3511. PMID: 19072749.
14. Berger A, Preiser W, Kachel HG, Stürmer M, Doerr HW. HBV reactivation after kidney transplantation. J Clin Virol. 2005 Feb;32(2):162-5. doi: 10.1016/j.jcv.2004.10.006. PMID: 15653420.
15. Duhart BT Jr, Honaker MR, Shokouh-Amiri MH, Riely CA, Vera SR, Taylor SL, et al. Retrospective evaluation of the risk of hepatitis B virus reactivation after transplantation. Transpl Infect Dis. 2003 Sep;5(3):126-31. doi: 10.1034/j.1399-3062.2003.00021.x. PMID: 14617300.
16. Blanpain C, Knoop C, Delforge ML, Antoine M, Peny MO, Liesnard C, Vereerstraeten P, Cogan E, Adler M, Abramowicz D. Reactivation of hepatitis B after transplantation in patients with pre-existing anti-hepatitis B surface antigen antibodies: report on three cases and review of the literature. Transplantation. 1998 Oct 15;66(7):883-6. doi: 10.1097/00007890-199810150-00012. PMID: 9798698.
