# Supplementary material for: Incidence, risk factors, and clinical outcomes of HBV reactivation in non-liver solid organ transplant recipients with resolved HBV infection: A systematic review and meta-analysis
Source: PLoS Med. 2023 Mar 15;20(3):e1004196. doi: 10.1371/journal.pmed.1004196 (PMC10058170; doi:10.1371/journal.pmed.1004196)
Supplement: S4 Table — (DOCX) [file pmed.1004196.s004.docx]

S4 Table: incidence of hepatitis b virus reactivation in non-liver solid organ transplant recipients.

| Subgroup | Study number | Events/Total patients | Proportion (95%CI) | Predication interval (95%) | I^2^ (95%CI) | Cochrane Q value | p value for heterogeneity |
| --- | --- | --- | --- | --- | --- | --- | --- |
| **Total** | 15 | 76/2913 | 0.025 (0.016-0.036) | 0.004-0.064 | 54.6% (18.7%-74.6%) | 30.83 | 0.006 |
| **Sex** |  |  |  |  |  |  |  |
| Female | 3 | 13/295 | 0.051 (0.015-0.109) | (0-0.968) | 60.7% (0.0%-88.8%) | 5.09 | 0.079 |
| Male | 3 | 16/528 | 0.031 (0.013-0.058) | (0.013-0.058) | 44.2% (0.0%-83.4%) | 3.59 | 0.166 |
| **Geographical location** |  |  |  |  |  |  |  |
| Asian population | 7 | 58/2201 | 0.028 (0.016-0.043) | (0.003-0.079) | 63.2% (16.7%-83.8%) | 16.30 | 0.010 |
| non-Asian population | 9 | 18/712 | 0.022 (0.009-0.039) | (0-0.077) | 44.9% (0.0%-74.5%) | 14.50 | 0.070 |
| **Transplantation type** |  |  |  |  |  |  |  |
| ABO blood type-compatible | 3 | 13/526 | 0.029 (0.008-0.071) | (0-0.671) | 56.0% (0.0%-87.4%) | 4.55 | 0.103 |
| ABO blood type-incompatible | 3 | 8/111 | 0.070 (0.029-0.127) | (0-0.657) | 0.0% (0.0%-89.6%) | 1.83 | 0.400 |
| **Anti-HBs status** |  |  |  |  |  |  |  |
| Negative | 6 | 34/421 | 0.078 (0.052-0.109) | (0.034-0.138) | 36.0% (0.0%-74.5%) | 7.82 | 0.170 |
| Positive | 6 | 27/1727 | 0.015 (0.010-0.021) | (0.008-0.024) | 0.0% (0.0%-74.6%) | 3.27 | 0.660 |
| **Use of anti-thymocyte globulin** |  |  |  |  |  |  |  |
| No | 4 | 13/764 | 0.017 (0.009-0.027) | (0.003-0.043) | 0.0% (0.0%-84.7%) | 0.69 | 0.880 |
| Yes | 4 | 25/504 | 0.049 (0.025-0.081) | (0-0.205) | 49.0% (0.0%-83.1%) | 5.88 | 0.120 |
| **Use of rituximab** |  |  |  |  |  |  |  |
| No | 5 | 13/675 | 0.017 (0.009-0.028) | (0.005-0.037) | 41.5% (0.0%-78.4%) | 6.83 | 0.144 |
| Yes | 3 | 10/133 | 0.073 (0.034-0.126) | (0-0.638) | 0.0% (0.0%-89.6%) | 1.75 | 0.418 |
| **History of acute rejection** |  |  |  |  |  |  |  |
| Yes | 3 | 13/266 | 0.058 (0.023-0.145) | (0-1.000) | 63.2% (0.0%-89.5%) | 5.43 | 0.066 |
| No | 3 | 19/735 | 0.027 (0.017-0.042) | (0.002-0.476) | 0.0% (0.0%-89.6%) | 1.35 | 0.510 |
| **Solid organ transplantation type** |  |  |  |  |  |  |  |
| Kidney | 12 | 65/2840 | 0.027 (0.018-0.038) | (0.0060.063) | 53.8% (11.3%-76.0%) | 23.80 | 0.011 |
| **Antiviral prophylaxis** |  |  |  |  |  |  |  |
| No | 9 | 49/1502 | 0.031 (0.019-0.046) | (0.006-0.077) | 48.0% (0.0%-74.9%) | 17.30 | 0.040 |
| Yes | 2 | 2/124 | 0.019 (0-0.080) | NA | 34.3% | 0.22 | 0.220 |

HBV: hepatitis b virus; CI: confidence interval; NA: not available
