# Supplementary material for: Incidence, risk factors, and clinical outcomes of HBV reactivation in non-liver solid organ transplant recipients with resolved HBV infection: A systematic review and meta-analysis
Source: PLoS Med. 2023 Mar 15;20(3):e1004196. doi: 10.1371/journal.pmed.1004196 (PMC10058170; doi:10.1371/journal.pmed.1004196)
Supplement: S5 Table — (DOCX) [file pmed.1004196.s005.docx]

S5 Table: Results of meta-regression analyses

| Variables | Study number | Odds ratio (95%CI) | p value |
| --- | --- | --- | --- |
| Age | 15 | 1.005 (1.000-1.010) | 0.053 |
| Sex (male, female) | 14 | 1.232 (0.805-1.886) | 0.336 |
| Publication year | 15 | 1.002 (0.996-1.007) | 0.563 |
| Country (Asian, non-Asian) | 15 | 0.988 (0.921-1.060) | 0.737 |
| Organ type (kidney, non-kidney) | 15 | 0.891 (0.785-1.012) | 0.077 |
| Sample size | 15 | 1.000 (1.000-1.000) | 0.707 |
| Follow-up duration | 12 | 1.000 (0.999-1.001) | 0.620 |
| ABO blood type-incompatible transplantation | 4 | 1.818 (0.546-6.054) | 0.330 |
| Rituximab | 7 | 1.406 (0.991-1.994) | 0.056 |
| Anti-HBs status (negative, positive) | 9 | 0.782 (0.645-0.949) | 0.013 |
| Anti-thymocyte globulin use | 6 | 1.047 (0.877-1.252) | 0.610 |
| Antiviral prophylaxis | 4 | 1.193 (0.952-1.496) | 0.126 |

95%CI: 95% confidence interval, anti-HBs: antibody against hepatitis b surface antigen
