# Supplementary material for: Incidence, risk factors, and clinical outcomes of HBV reactivation in non-liver solid organ transplant recipients with resolved HBV infection: A systematic review and meta-analysis
Source: PLoS Med. 2023 Mar 15;20(3):e1004196. doi: 10.1371/journal.pmed.1004196 (PMC10058170; doi:10.1371/journal.pmed.1004196)
Supplement: S6 Table — (DOCX) [file pmed.1004196.s006.docx]

S6 Table: Meta-analyses of hepatitis b virus-related complications

| Complications | Study number | Events/Total patients | proportion (95%CI) | Predication interval (95%) | I^2^ (95%CI) | Cochrane Q value | p value for heterogeneity |
| --- | --- | --- | --- | --- | --- | --- | --- |
| Impaired liver function | 8 | 21/45 | 0.331 (0.047-0.714) | (0-1.000) | 82.4% (66.6%-90.7%) | 39.80 | <0.001 |
| Hepatic cirrhosis | 7 | 8/26 | 0.189 (0.017-0.484) | (0-0.957) | 59.7% (7.3%-82.5%) | 14.88 | 0.012 |
| Hepatic failure | 8 | 7/52 | 0.110 (0.040-0.208) | (0.001-0.267) | 0.1% (0.0%-67.6%) | 7.00 | 0.428 |
| HBV-related death | 8 | 7/52 | 0.110 (0.040-0.208) | (0.001-0.267) | 0.1% (0.0%-67.6%) | 7.00 | 0.428 |

HBV: hepatitis b virus; CI: confidence interval
