# Supplementary material for: Incidence, risk factors, and clinical outcomes of HBV reactivation in non-liver solid organ transplant recipients with resolved HBV infection: A systematic review and meta-analysis
Source: PLoS Med. 2023 Mar 15;20(3):e1004196. doi: 10.1371/journal.pmed.1004196 (PMC10058170; doi:10.1371/journal.pmed.1004196)
Supplement: S7 Table — (DOCX) [file pmed.1004196.s007.docx]

S7 Table: Risk factors of hepatitis b virus reactivation in non-liver solid organ transplant recipients with resolved hepatitis b virus infection.

| Variables | Study number | Models | Events/Total number | Odds ratio(95%CI) | p value | I2(95%CI) | Predication interval (95%) | Cochrane Q | p value for heterogeneity |
| --- | --- | --- | --- | --- | --- | --- | --- | --- | --- |
| Age (≥60 vs <60 years) | 2 | Random-effects | 8/127 vs 16/672 | 2.39 (0.11-53.21) | 0.580 | 83.3% (30.7%-96.0%) | NA | 6.00 | 0.014 |
| Sex (Male vs Female) | 3 | Random-effects | 16/528 vs 13/295 | 0.67 (0.31-1.42) | 0.294 | 0.0% (0.0%-89.6%) | (0.01-91.11) | 0.50 | 0.753 |
| Donors' anti-HBc status (negative vs positive) | 2 | Random-effects | 5/245 vs 10/420 | 0.88 (0.30-2.62) | 0.820 | 0% | NA | 0.42 | 0.518 |
| Anti-HBs status (negative vs positive) | 6 | Random-effects | 34/421 vs 27/1727 | 5.05 (2.83-9.00) | <0.001 | 0.0% (0.0%-74.6%) | (2.23-11.44) | 4.08 | 0.539 |
| Anti-HBs level (>100 IU/L vs 10-100 IU/L) | 1 | NA | 1/113 vs 3/96 | 0.28 (0.03-2.71) | 0.270 | NA | NA | NA | NA |
| ABO-incompatible vs ABO-compatible transplantation | 3 | Random-effects | 8/111 vs 13/526 | 2.62 (1.05-6.04) | 0.040 | 0.0% (0.0%-89.6%) | (0.01-997.76) | 0.61 | 0.737 |
| Anti-thymocyte globulin use (Yes vs No) | 4 | Random-effects | 25/504 vs 13/764 | 3.19 (1.48-6.87) | 0.003 | 0.0% (0.0%-84.7%) | (0.59-17.18) | 1.39 | 0.661 |
| Rituximab use (Yes vs No) | 3 | Random-effects | 10/133 vs 11/540 | 3.16 (1.24-8.06) | 0.016 | 0.0% (0.0%-89.6%) | (0.01-1372.82) | 1.69 | 0.429 |
| Rituximb dose: 375mg/m2 vs 200mg/body | 1 | NA | 5/57 vs 0/34 | 7.23 (0.39-134.94) | 0.185 | NA | NA | NA | NA |
| Acute rejection | 3 | Random-effects | 13/266 vs 19/735 | 2.37 (1.13-4.97) | 0.022 | 0.0% (0.0%-89.6%) | (0.02-284.46) | 1.07 | 0.586 |
| Antiviral Prophylaxis (Yes vs No) | 2 | Random-effects | 2/124 vs 19/359 | 0.51 (0.03-8.17) | 0.637 | 70.3% (0.0%-93.3%) | NA | 3.36 | 0.067 |

HBV: hepatitis b virus; NA: not available; 95%CI: 95% confidence interval
