# Supplementary material for: Incidence, risk factors, and clinical outcomes of HBV reactivation in non-liver solid organ transplant recipients with resolved HBV infection: A systematic review and meta-analysis
Source: PLoS Med. 2023 Mar 15;20(3):e1004196. doi: 10.1371/journal.pmed.1004196 (PMC10058170; doi:10.1371/journal.pmed.1004196)
Supplement: S1 Data — (ZIP) [file pmed.1004196.s010.zip › Raw meta data/Risk factors of HBV reactivation/R code for Risk factors of HBV reactivation.docx]

R code for **Risk factors of HBV reactivation**

bmi_rc <- read.csv("rituximab dose.csv", sep=",", header=T)

bmi_rc

library("meta")

metabmi_rc=metabin(a,n1,c,n2,data = bmi_rc,sm="OR", studlab = study)

metabmi_rc

forest(metabmi_rc)

funnel(metabmi_rc)
