# Supplementary material for: Incidence, risk factors, and clinical outcomes of HBV reactivation in non-liver solid organ transplant recipients with resolved HBV infection: A systematic review and meta-analysis
Source: PLoS Med. 2023 Mar 15;20(3):e1004196. doi: 10.1371/journal.pmed.1004196 (PMC10058170; doi:10.1371/journal.pmed.1004196)
Supplement: S1 Data — (ZIP) [file pmed.1004196.s010.zip › Raw meta data/The incidence of HBV reactivation/R code for incidence of HBV infection.docx]

R code for incidence of HBV infection

bmi_rc <- read.csv("non-Asia.csv", sep=",", header=T)

bmi_rc

library("meta")

metarate = metaprop(event, n, studlab = study, data = bmi_rc, sm = "PAS", incr=0.5, allincr=FALSE, addincr=FALSE, comb.fixed=FALSE, comb.random=TRUE, print.byvar=TRUE,warn=TRUE)

metarate

forest(metarate,digits = 3)

funnel(metarate)

inf <- metainf(metarate)

inf

forest(inf)
